# 61 The nailfold videocapillaroscopy in pediatric Behçet’s disease: a multi-center study

**DOI:** 10.1093/rheumatology/keac496.057

**Published:** 2022-10-07

**Authors:** Figen Çakmak, Şeyda Doğantan, Rana İşgüder, Müşerref Kasap Cüceoğlu, Şengül Çağlayan, Hafize Emine Sönmez, Özlem Akgün, Betül Sözeri, Ayşenur Paç Kısaarslan, Erbil Ünsal, Seza Özen, Nuray Aktay Ayaz

**Affiliations:** 1 Istanbul University, Istanbul Faculty of Medicine, Pediatric Rheumatology; 2 Erciyes University, Faculty of Medicine Pediatric Rheumatology; 3 Dokuz Eylül Faculty of Medicine, Pediatric Rheumatology; 4 Hacettepe University, Faculty of Medicine, Pediatric Rheumatology; 5 Ümraniye Health and Science Hospital, Pediatric Rheumatology; 6 Kocaeli University, Istanbul Faculty of Medicine, Pediatric Rheumatology

## Abstract

**Background:**

Behçet's disease (BD) is a chronic inflammatory disease characterized by recurrent oral aphthous and genital ulcers accompanied by eye, joint, skin, gastrointestinal and central nervous system involvement. The vascular involvement may affect both the arterial and venous systems. Nailfold videocapillaroscopy (NVC) is an easy and non-invasive method used in the evaluation of microcirculation. With this study, we aimed to find the characteristics and prevalence of nailfold capillary alterations in patients with juvenile BD and to analyze their possible relationship between clinical characteristics and activity of the disease.

**Methods:**

Patients aged 5–21 years with a diagnosis of juvenile BD and followed up for at least six months were included in the study. Demographic and clinical characteristics of the patients were recorded. NVC was performed on 8 fingers of both hands, excluding the thumbs, and four consecutive non overlapping fields for each of fingers were evaluated (32 fields per patient). Capillary density, capillary width (arterial width, venous width, apical loop), capillary morphology and the presence of meandering capillary, micro hemorrhage, avascular area, neoangiogenesis, capillary ramification were evaluated from the images. Capillary morphology were evaluated by classifying them into four groups as normal, minor abnormalities, major abnormalities and scleroderma pattern. The presence of abnormilities in at least two fingers were recorded as capillary abnormality. The semi quantitative rating score 1–3 was applied for each capillaroscopic alteration.

**Results:**

37 patients from 6 pediatric rheumatology centers were included in the study. The mean age of patients was 17 years (IOR 13–19) and 20 (54.1%) of them were girls. The patients were evaluated in four clusters according to their clinical presentations. Nineteen patients had mucocutaneous involvement, 9 patients had uveitis, 8 patients had vascular and neurological involvement, and 4 patients had gastrointestinal system involvement. During the follow-up period, genital ulcers developed in 22 patients, erythema nodosum in 9 patients, pseudofolliculitis in 18 patients, uveitis in 10 patients, vascular involvement in 8 patients, and neurological involvement in 5 patients. Anterior uveitis was present in five, posterior uveitis in three, panuveitis in one, and retinal vasculitis in three of the patients with ocular involvement. Four patients had lower extremity venous thrombosis, three patients had central nervous system (CNS) thrombosis, and one patient had both lower extremity and CNS thrombosis.

When capillary morphology was evaluated; normal morphology was present in 16 patients, minor abnormality in 13 patients, and major abnormality in 8 patients. Median capillary density was 8, capillary length was 325 µm, arterial width was 12 µm, venous width was 16 µm, apical loop width was 18 µm, capillary width was 39 µm, and intercapillary distance was 107 µm. Neoagiogenesis was seen in 13 patients, enlarged capillaries in 12 patients, capillary meandering in 9 patients, bushy capillaries in 5 patients, bizarre capillaries in 4 patients, and microhemorrhage in 3 patients. Neoangiogenesis was found to be significantly more common in the NVC evaluation of patients with lower haemoglobin values at the time of diagnosis (*p* = 0.014).

**Conclusion:**

NVC is an *in vivo*, non-invasive, and inexpensive imaging technique that allows the direct observation of the capillary network in living tissue throughout the skin and it may be preferred in juvenile BD for evaluating microvascular involvement.

**61 Figure 1:**
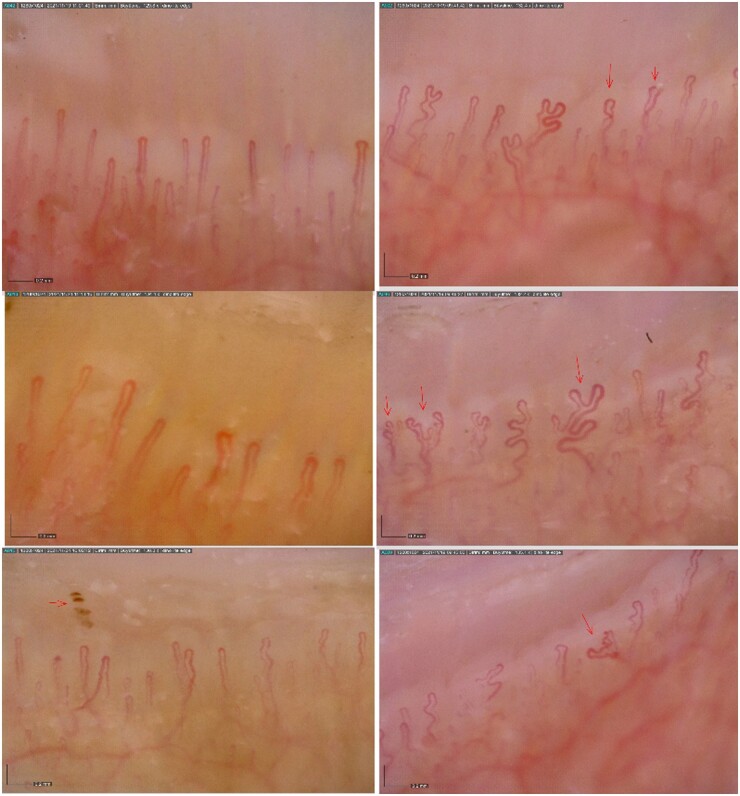
Capillaroscopic alterations of patients

